# Compressibility of expansive soil mixed with sand and its correlation to index properties

**DOI:** 10.1016/j.heliyon.2024.e35711

**Published:** 2024-08-03

**Authors:** Ammar Alnmr, Rashad Alsirawan, Richard Ray, Mounzer Omran Alzawi

**Affiliations:** a Department of Structural and Geotechnical Engineering, Faculty of Architecture, Civil Engineering and Transport Sciences, Széchenyi István University, Egyetem tér 1, 9026, Győr, Hungary; b Department of Geotechnical Engineering, Faculty of Civil Engineering, Tishreen University, Lattakia, Syria

**Keywords:** Expansive soil, Sand, Oedometer test, Partial saturation, Initial dry unit weight

## Abstract

Prior research has primarily focused on Atterberg limits, void ratios, and/or water content, often disregarding the impact of coarse material percentage in the soil, which significantly affects compressibility behavior. This paper examines the effects of sand content, initial degree of saturation, and initial dry unit weight on the compressibility behavior of expansive soils. Ninty-six oedometer tests were performed in order to accurately predict the compressibility behavior of expansive soils. The previous studies have attempted to correlate compressibility with different index properties separately, but no single study has taken into consideration all properties influencing compressibility behavior, especially for expansive soils. The findings show that compressibility is greatly influenced by the sand content, initial degree of saturation, and initial dry unit weight. Increasing the initial dry unit weight specifically lowers the compression index and permeability while raising the recompression index for the same percentage of added sand. Moreover, since swelling reduces with increasing initial saturation, raising the saturation degree also lowers the permeability, recompression index, and compression index. The results indicate that a sand content of more than 30 % is recommended for achieving desired properties in expansive clayey soil. This is a result of sand taking the dominant role in the soil mixture, which lowers soil suction and improves soil properties by reducing swelling, permeability, and compressibility. Symbolic regression equations were created to predict the compression and recompression indices, outperforming previous models in accurately predicting the compressibility behavior of expansive soils, considering the percentage of sand. The validation of these equations demonstrates their predictive capabilities.

## 1 Introduction

Expansive clayey soils cover vast areas of land throughout the world. They are a significant and costly problem for engineered infrastructure, resulting in billions of dollars of housing and building repair, roadway and railway maintenance, and replacement and renovation of lifelines [[1], [2], [3], [4]]. According to Nelson and Miller [5], the financial loss caused by expansive soils is more significant than that caused by earthquakes or floods. Designing and building on expansive soils requires understanding their complex behavior and how they interact with the environment. Reducing their damaging impact requires intervention by removing the problematic soil, treating it with cement, lime or pozzolanic materials [[6], [7], [8]], or mixing it with non-plastic sand [9,10] to produce a more stable and predictable soil support skeleton. The critical aspects of sand mixing focus on the optimum quantity of sand added and the conditions for compacting the clay-sand mixture.

### 1.1 Sand as an additive material

Several researchers, Louafi & Bahar [11], Kaoua et al. [12], Nagaraj [13], Phanikumar et al. [14], Deng et al. [15], Elmannaey et al. [16], and Alnmr et al. [17] added different materials to expansive soils to study their behavior. Although these materials enhance the properties of expansive soils, many questions about the resulting mix's behavior require further investigation. Experimental studies were conducted by Louafi & Baher [11], Roy [18], Srikanth & Mishra [19], and Atemimi [20] to study the effect of sand on soil plasticity. Their results showed that adding sand to clayey soil significantly reduced the plasticity index. Their reason for improving clayey soil properties was that sand particles replaced soft soil particles in the structural matrix within a significant portion of the sample. As a result, the soil will have greater bearing capacity and less settlement when adequately mixed and compacted on site.

Many previous studies investigated the effect of sand on the compressive behavior of expansive soils, but some of these studies were limited to specific percentages of sand or distant percentages, and most studies did not consider all three factors: sand percentage, initial dry unit weight, and level of saturation on the compressive behavior of expansive soils. These factors are regarded as critical in understanding the behavior of expansive soils, particularly since these soils are extremely sensitive to water and have a significant influence on them. These three characteristics influence the soil's ability to absorb water, which in turn affects its compressive behavior. Matric suction increases as the percentage of added sand decreases, and it also increases when the initial dry unit weight increases. However, after adding 20 % sand, the water-holding capacity significantly decreases. Furthermore, when the initial degree of saturation approached 90 %, the typical curves matched in terms of volumetric water content, which decreased as the degree of saturation increased. Table 1 summarizes several research that studied the effect of sand content, initial dry unit weight and initial degree of saturation on the oedometer test of clayey soils [21,22].

**Table 1:** Overview of the research that studied the effect of sand content, initial degree of saturation and initial dry unit weight on the oedometer test of expansive soils.

Reference	Studied parameters	Description
[9]	Sand (0–25 %)	This study looks at how adding sand affects the compressibility and permeability of bentonite-sand mixtures, finding that as sand content increases, swelling and swelling pressure decrease. The study emphasizes changes in consolidation parameters and demonstrates a linear relationship between void ratio and permeability as sand content increases.
[3]	sand and Class C fly ash used (0–50 %)	This research looks at how Ottawa sand and Class C fly ash affect the swelling characteristics and expansive index of bentonite and kaolinite clay. Swelling was reduced by 10–50 % for kaolinite and 4–49 % for bentonite, with a 64 % reduction in swelling pressure for bentonite and 93 % for kaolinite. The study also used standard index and consolidation tests to compare the properties of these mixtures to pure clay.
[23]	Dry unit weight, and water content	In this study, oedometer tests on German bentonite revealed a relationship between swelling pressure and initial water content and dry density. Initial compaction influenced compression index, but the effects on the recompression index were found to be less significant. At higher void ratios, the coefficient of consolidation varied significantly, impacting permeability differently in saturated and compacted specimens.
[10]	Sand (7–49 %), dry unit weight, and water content	This study looked at estimating the recompression index (swell index). It reviewed common empirical equations using only 42 test data points and introduced improved equations. Furthermore, an artificial neural network with specific parameters was developed to predict the swell index, outperforming traditional equations. However, due to the limited data set, it may not accurately capture the behavior.
[24]	Dry unit weight, and water content	A GMDH neural network is used in this study to estimate *Cr* using *LL*, *e*_*0*_, and *Gs*. The model incorporates *w*_*n*_, *PI*, and γ d, along with these properties, and is based on 344 case history data sets from Mazandaran, Iran.
[25,26]	Dry unit weight, and water content	These studies propose an equation for saturated fine-grain soils that links the virgin compression index to the slope of the instantaneous zero-air-voids curve. Based on field data, it assumes parallel consolidation curves at certain void ratios and depicts soil processes with the same specific gravity with a single curve.
[27]	Dry unit weight, and water content	Regression models were guided by consolidation tests on Korean coastal marine clay. Unlike previous empirical formulations, a simple linear equation based on water content, void ratio, and liquid limit effectively predicts compression index, revealing significant uncertainty in this soil's prediction.
[28]	Dry unit weight, and water content	This study used molecular dynamics (MD) simulations to calculate the bulk modulus of montmorillonite at varied water levels and dry densities. The results reveal that dry density and water content have a significant influence on the microscopic bulk modulus.
[29]	Dry unit weight	Over 700 consolidation experiments on varied undisturbed soils were statistically investigated in this study. To estimate the compression index and recompression index, regression equations were created. These equations were compared to other empirical relationships, revealing a reliable linear regression model for estimating compression parameters using the initial void ratio.
[30]	Dry unit weight	This study investigated the development of one-dimensional volumetric strain during soaking and wetting in expanding clay from Nanyang, China. Different initial densities and vertical stress levels were examined in the study. It was discovered that under low vertical stress, high initial densities caused swelling, which changed to collapse as stress increased and dry density decreased. The initial dry density has a major effect on the volumetric change.

### 1.2 Soil compressibility

**Fig. 1:**
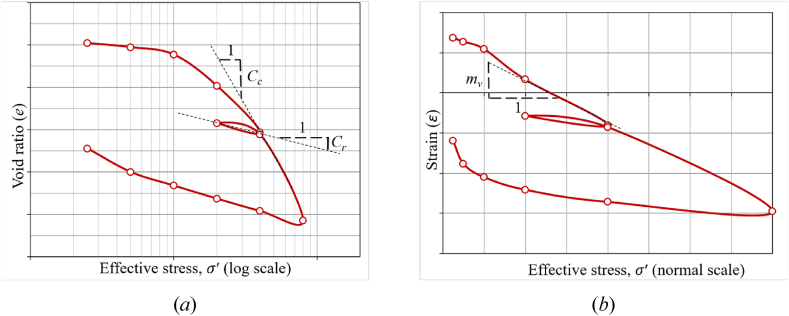
Typical one-dimensional consolidation curves that illustrating the parameters (*a*) $C_{c}\;\text{and}\;C_{r}$, (*b*) ${\;m}_{v}$.

Soil compressibility is a critical engineering consideration for soils. Granular soils derive their compressive stiffness from their density, i.e., the arrangement of individual particles. Clay soils interact more with water and dissolved ions [28,31,32], so particle arrangements from the sub-millimeter to the nanometer scale influence their behavior. Geotechnical engineers quantify their mechanical behavior by loading them in confined compression (oedometer) and recording the resulting displacement. Generally, clays exhibit deformation proportional to the logarithm of applied loading (log *P*). The slope of the log *P* vs deflection (strain *εv*, void ratio, *e*) represents the clay's compressibility. The compression index ($C_{c}$) represents clay stiffness during initial loading. The recompression index ($C_{r}$) represents stiffness during unloading and reloading back to a previous load level. Both $C_{c}$ and $C_{r}$ are calculated as the slope of the linear part of the void ratio, *e* vs. the logaritham of effective stress ***σ***′ (log ***σ***′). Recompression is generally stiffer than initial compression. The coefficient of volume compressibility ($m_{v}$) represents clay stiffness. However it is calculated as the slope of the linear part of the strain, ε vs. the effective stress ***σ***′. An additional term, coefficient of consolidation ($C_{v}$), approximates the time for 50 % consolidation for a given load increment. Calculating hydraulic conductivity (Permeability) through consolidation tests is dependent on $C_{v}$ and $m_{v}$ values. The compression and recompression indices predict how much settlement will take place, while the coefficient of consolidation predicts the time required to achieve that settlement. Fig. 1 depicts a one-dimensional consolidation curve that depicts the consolidation behavior of the sample during the loading sequence. Fig. 1(a) shows the correlation between (*e* vs log ***σ***′), which aids in the computation of $C_{c}$ and $C_{r}$. Fig. 1(b) also shows the correlation between (*ε* vs ***σ***′), which is used to calculate $m_{v}$.

A laboratory oedometer test will measure $C_{c}$, $C_{r}$, and $c_{v}$, but requires days or weeks to complete. To reduce the time required to obtain valid estimates of compressibility, engineers have developed correlations between the oedometer test results and simpler index tests that they perform in hours. Numerous authors have predicted $C_{r}$ and $C_{c}$ from other index properties that can measured more readily. Skempton [33] presented the first well-known correlation for $C_{c}$ as a function of liquid limit (*LL*) for remolded clays. Other researchers have proposed similar correlations for $C_{c}$ and $C_{r}$ using different index properties suitable for different clay classifications. These equations appear in Table 2 for $C_{c}$ and 3 for $C_{r}$.

**Table 2:** Summary of the regression equations of $C_{c}$ mentioned in previous research.

Equation	R^2^	Reference	Suitability
$C_{c}$ = 0.007(***LL*** -10)	–	Skempton 1944 [33]	Remolded clays
$C_{c}$ = 0.2237***LLG***_***s***_	–	Nagaraj and Srinivasa 1983 [34]	All remolded, normally consolidated clays
$C_{c}$ = 0.29(***e***_***0***_-0.27)	0.408	Hough 1957 [35]	Inorganic, cohesive soils
$C_{c}$ = 0.009(***LL***-10)	0.424	Terzaghi and Peck 1967 [36]	Normally consolidated clays
$C_{c}$ = 0.37*[ ***e***_***0**+*_ 0.003 ***LL***+ 0.0004 $w_{n}$ −0.34]	0.74	Azzouz et al., 1976 [29]	All clays with LL < 100 %
$C_{c}$ = 0.256 + 0.00106*(***LL*** -0.65)+0.32*($e_{0}$-0.84)+0.063	–	Cozzolino 1961 [37]	Lowlands of Santos, Brazil
$C_{c}$ = 0.156* $e_{0}$ +0.0107	0.3157	Bowles 1989 [38]	All clays
$C_{c}$ = 0.01* $w_{n}$	0.52	Koppula 1981 [39]	All clays
$C_{c}$ = 0.141 $G_{s}{\left(\gamma_{w}/\gamma_{d}\right)}^{2.4}$	0.85	Herrero 1980 [26]	All soil types
$C_{c}$ = 0.01($w_{n}$ −7.549)	0.94	Herrero 1983 (1) [25]	All soil types
$C_{c}$ = 0.3745 $e_{0}$	0.52	Herrero 1983 (2) [25]	All soil types
$C_{c}$ = 0.5 ***PIG***_***s***_	–	Wroth and Wood 1978 [40]	All remolded, normally consolidated clays
$C_{c}=e_{0}+\left[\left(e_{0}+2LL\right)/\left(e_{0}-6.87\right)\right]\;\left[-0.35+{LL}^{2}\right]+{\left[\log\left(2e_{0}+2LL-2PL+0.15\right)\right]}^{2}$	0.832	Mohammadzadeh et al., 2019 [41]	Lean clay
$C_{c}=exp\left\{-21.567+18.227\;ln\left(w_{n}\right)-0.743{\left[ln\left(w_{n}\right)\right]}^{2}\right\}$	–	Löfman & Korkiala-Tanttu. 2021 [42]	Finnish clays

Table 2, Table 3 present the correlation equations of compression and recompression indices, respectively, illustrating parameters used by various researchers. These include liquid limit (LL), plasticity index (PI), initial void ratio ($e_{0}$), dry unit weight ($\gamma_{d}$), and natural moisture content ($w_{n}$). Note that these equations do not account directly for the effect of sand, which significantly impacts volume-change properties. Table 2, Table 3 show that the majority of the research used empirical prediction equations obtained from linear or multiple linear regression analysis. Because of the inherent simplicity of the model, the use of regression analysis includes certain restrictions and uncertainties. Traditional regression approaches, such as linear and multiple regression, presume a predefined relationship between the input and result. This assumption sets constraints on the model's adaptability and may cause potential problems in capturing complicated relationships within the data. Given the abundance of equations in Table 2, Table 3, a comparison between them is required to determine whether they predict the real behavior of expansive soil. Some equations do not have enough features to capture precisely the correct behavior, and this comparison is done later in this study in section 3.3. Furthermore, researchers have investigated the applicability of various machine learning (ML) models for forecasting the compression parameters [[43], [44], [45], [46], [47], [48]]. The results show that artifitial neural network (ANN) models have distinct prediction capabilities and show good potential in calculating the compression parameters. This demonstrates how, when compared to traditional empirical formulas or regression-based methods, machine learning methods improve prediction accuracy and performance. However, despite their high performance, artifitial newral network (ANN), random forest regression (RFR), gradient boosting regression (GBR), support vector regression (SVR), and other ML approaches are considered black-box models since they are unable to develop effective prediction equations, which is a significant constraint in their applicability [49]. To address these shortcomings, a robust ML technique, symbolic regression (SR), based on genetic programming (GP), was developed [50]. Mohammadzadeh et al. [41] used this algoriths to predict compression index ($C_{c}$). However the study is for clayey soils classified as Lean clay (CL) and only three input parameters considered in their study ($e_{0}$, LL, and PL). In this study, symbolic regression machine learning will be used for the first time to predict both $C_{c}$ and $C_{r}$ of expansive soils. This method is capable of generating accurate equations that are directly applicable in practical scenarios, which is a novel approach in $C_{c}$ and $C_{r}$ predictions.

**Table 3:** Summary of the regression equation $C_{r}$ mentioned in previous research (compiled from Kordnaeij et al. [24]).

Equation	R^2^	Reference	Suitability
$C_{r}$ = 0.126($e_{0}$ +0.003***LL***-0.06)	0.58	Azzouz et al., 1976 (1) [29]	All clays (LL < 100 %)
$C_{r}$ = 0.014($e_{0}$ +0.007)	0.55	Azzouz et al., 1976 (2) [29]	All clays (LL < 100 %)
$C_{r}$ = 0.045–0.0283 $e_{0}$	–	Gunduz and Arman. 2007 [51]	Low-plasticity clayey soil
$C_{r}$ = 0.00084 (***PI***-4.6)	0.884	Nakase et al., 1988 [52]	Low and high plasticity clayey soil
$C_{r}$ = 0.0007 ***LL***+0.0062	0.291	Sinan. 2009 (1) [10]	All clays
$C_{r}$ = 0.1257 ***γ***_***d***_^−2.8826^	0.6532	Sinan. 2009 (2) [10]	All clays
$C_{r}$ = 0.0133*e*^−0.036^***^wn^***	0.6187	Sinan. 2009 (3) [10]	All clays
$C_{r}$ = 0.000463 ***LL G***_***s***_	0.3418	Nagaraj and Murty. 1985 [53]	All clays
$C_{r}$ = −0.000319 $w_{n}$ −0.027277 ***γ***_***d***_ +0.064019 ***e***_***0***_ +0.037	0.6857	Sinan. 2009 (4) [10]	All clays
$C_{r}$ = - 0.0115 + 0.587***Y***_***1***_+ 0.00017 ***LL*** + 0.524 ***Y***_***1***_^2^ + 0.000008 ***LL***^2^ + 0.0015 ***LL******Y***_***1***_ ***Y***_***1***_ = -1.689 + 0.125 $e_{0}$ +1.286 ***G***_***s***_ −0.027 $e_{0}$^*2*^ -0.251 ***G***_***s***_^2^ + 0.00004 $e_{0}$***G***_***s***_	0.956	Kordnaeij et al., 2015 [24]	All clays
$C_{r}=exp\left\{-12.339+3.618\;ln\left(w_{n}\right)-0.302{\left[ln\left(w_{n}\right)\right]}^{2}\right\}$	–	Löfman & Korkiala-Tanttu. 2021 [42]	Finnish clays

### 1.3 Research scope

Numerous studies have demonstrated that adding sand can improve the characteristics of expansive soil, offering an economically viable alternative to traditional methods. However, much existing research has not properly investigated the effect of sand content, saturation level, and dry unit weight on oedometer test findings in expansive soils. Specifically, the effect of sand content on permeability (hydraulic conductivity) has received little attention, with most research only taking modest amounts of sand. Furthermore, prior research has not presented predictive equations for evaluating the mechanical properties of expansive soil with variable sand content. This study fills these gaps by performing a variety of tests on expansive soils, including Atterberg limits, oedometer tests with varying initial conditions, and Proctor tests, providing a dataset of 96 records with 5 input features and 2 labels. Symbolic regression (SR) was utilized to create predictive equations for compression and recompression indices, which were then meticulously evaluated and compared.

This research advances soil mechanics by using symbolic regression to predict compression and recompression indices while controlling for sand content. This method provides a complete understanding of how variables like sand content, saturation levels, and dry densities affect expansive soil behavior. The novelty of the methodology is that it uses symbolic regression to assess raw experimental data, resulting in precise predictions of soil characteristics. This approach is notable for taking into account sand content, which was frequently disregarded in previous research. By including this feature, the prediction equations provide a more thorough understanding of soil behavior, bridging a substantial gap in the literature and increasing real-world applicability. Given the distinct features and behaviors of expansive soils, which necessitate specific prediction models, this study advances geotechnical engineering by filling identified gaps. The use of symbolic regression and the inclusion of parameters such as sand content improve prediction reliability and accuracy, furthering the understanding of expansive soil behavior and establishing the framework for future field studies.

## 2 Research materials and methodology

### 2.1 Outline of methodology

The methodology entails using laboratory and symbolic regression machine learning methods to predict compressibility parameters ($C_{c}$ and $C_{r}$). The procedure includes conducting of all necessary physical and mechanical experiments, as well as the analysis and discussion of the results. A dataset of 96 output parameters ($C_{c}$ and $C_{r}$) and input parameters (*LL, Fs, e*_*0*_*, SR,* $\gamma_{d}$) is created for predictive model development. These specific input parameters were chosen due to their ease of obtaining through simple tests and their extensive use in earlier studies. After developing predictive models with the symbolic regression technique, the models' performance is assessed using metrics such as coefficient of determination (R^2^), mean absolute error (MAE), and root mean square error (RMSE). Finally, the predictive models are contrasted with earlier models to highlight any discrepancies.

### 2.2 Materials and laboratory tests

**Fig. 2:**
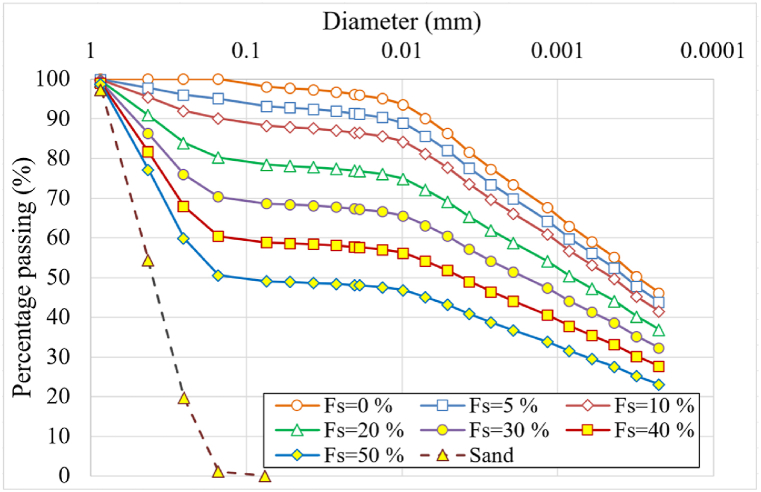
Grain size distribution for the clay and sand components and mixtures.

Field crews retrieved clayey soil samples from Demsarkho, Latakia, and the sand from the coastal region nearby. The sand used in this study is fine, chemically inert, marine siliceous sand that has been washed to remove impurities and fines before being mixed with the expansive soil. The mixing will lead to a change in the structural matrix of the mixture without any chemical reactions occurring. The mechanical characteristics at 50 % relative density are: ∅ = $39^{o}$, *c* = 0 kPa, $E_{oed}$ = 27.4 MPa. The morphology of the sand is described as fine-grained, angular, coarse-textured, and light brown in color. The sand was washed through a No.200 sieve to remove fines. Likewise, the washing process separated the Latakia clay from any coarser materials. After washing, the clay contained about 74 % particles smaller than 0.002 mm in diameter. Sand-clay mixtures were prepared according to dry weight, with sand percentages (0, 05, 10, 20, 30, 40, 50 %). Laboratory tests were carried out in compliance with ASTM standards. Grain Size Distribution measurements according to ASTM D6913 [54] and ASTM D7928-17 [55] produced the grain size curves shown in Fig. 2. Chemical composition tests (ASTM C311 [56]) produced an average mineral content shown in Table 4. Specific gravity testing (ASTM D854-14 [57]) generated results listed in Table 5.

**Table 4:** Chemical properties of clay fraction.

Chemical Composition	%
Alumina (Al_2_O_3_)	11.51
Ferric (Fe_2_O_3_)	5.49
Calcium (CaO)	12
Magnesium (MgO)	2.41
Silica (SiO_2_)	49.79
(Sodium (Na_2_O)	1.2
Potassium (K_2_O)	0.37
Loss of ignition (LoI)	17.23

**Table 5:** Physical and mechanical characteristics of clay and sand components.

Clay		Sand	
Parameter	Value	Parameter	Value
Liquid limit; *LL* (%)	79	Uniformity Coefficient *Cu* (−)	2.31
Plastic index; *PI* (%)	44	Curvature coefficient; *Cc* (−)	1.03
Shrinkage limit; *SL* (%)	12	Maximum void ratio; *e*_max_ (−)	0.844
		Minimum void ratio; *e*_min_ (−)	0.585
Permeability; *K* (m/day)	9.4 × 10^−07^		1.24
Specific gravity; *G* (−)	2.70		2.65
percentage passing the No. 200 sieve; (%)	100		0
USCS classification (ASTM D2487-17e1)	High plasticity clay (CH)		Poorly graded sand (SP)

**Fig. 3:**
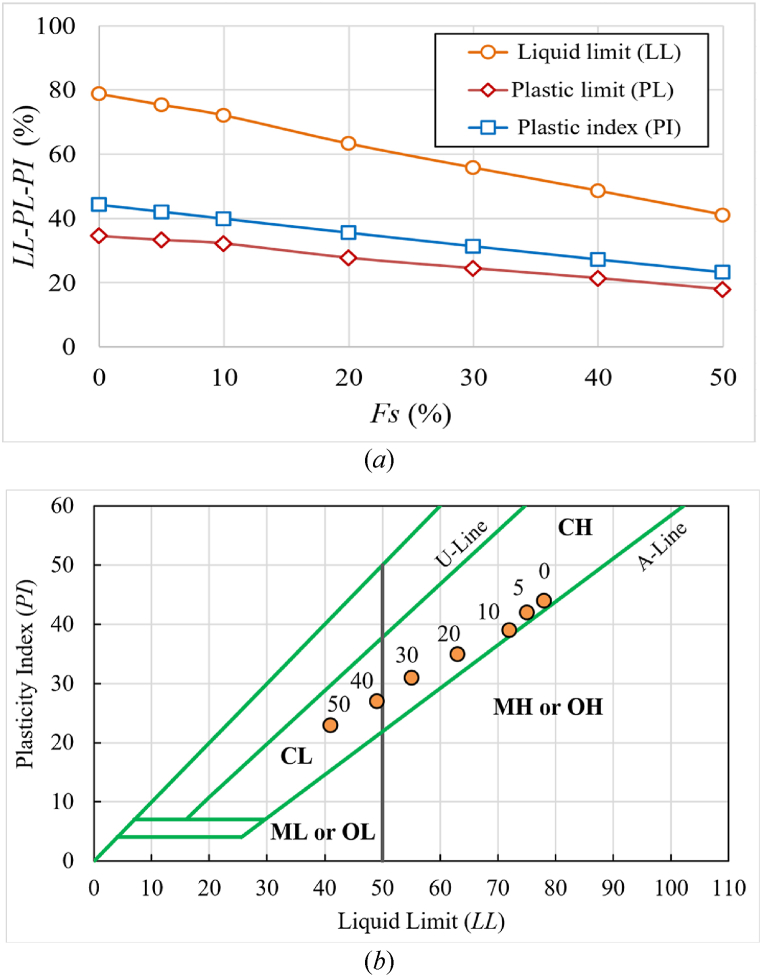
***a*)** Effect of percentages of sand on Atterberg limits, ***b*)** Clay samples position within the Casagrande's plasticity chart [61].

Furthermore, Atterberg limits for all mixtures were determined according to ASTM D4318-17e1 [58]. Fig. 3(a) shows the effect of varying percentages of sand on the Atterberg limits. Other experimental work on soil improvement with sand has yielded similar results [20,59]. Refer to Alnmr and Ray [60] for detailed information on Atterberg tests.

The AASHTO classification system [62] considers this clayey soil in the A-7-5 group and the USCS [63] places it in the CH category.

The clay mineralogy of soil was also represented by the Atterberg limits plot on a plasticity chart (Holtz & Kovacs [64]; Casagrande [61]). The Atterberg limits *LL* and *PI* for varying percentages of added sand were represented on a plasticity chart. The intersection points of *LL* and *PI* for each percentage of added sand were observed on Casagrande's plasticity chart, situated near or above the A-line, as illustrated in Fig. 3(b). According to Refs. [65,66], and [67], the degree of volume change was found to be Medium to very high as shown in Table 6. When the used expansive soil was prepared using standard proctor parameters ($\gamma_{d}$ = 13.95 kN/m^3^ and *SR* = 91.2 %), the swelling pressure was found to be nearly 200 kPa, whereas when the expanded soil was prepared using $\gamma_{d}$ = 15.3 kN/m^3^ and *SR* = 75 %, it was found to be nearly 520 kPa.

**Table 6:** The Degree of volume change.

Sand percentage (*Fs* %)	U. S. Bureau of Reclamation [65]	Raman 1967 [66]	Dakshanamurthy and Raman [67]
0	High to very high	Very high	Very high
10	High	Very high	Very high
20	Medium to high	Very high	High
30	Medium to high	High	High
40	Medium	High	Medium
50	Medium	Medium	Medium

The Prakash & Sridharan [68] method determined free swell for pure clay (without any sand content) as 127 %. The zero-swell consolidation test resulted in a swelling pressure of 255 kPa.

The standard Proctor Method [69] determined the components and mixtures’ maximum dry unit weight and optimum moisture content. The hydraulic conductivity (Permeability) resuleted from back-calculating using the time to 50 % consolidation during an oedometer test (ASTM D2435/D2435M − 11 [70]) via Eq. (1).

(1) $$k=c_{v}m_{v}\gamma_{w}$$

where *k* is the permeability, $\gamma_{w}$ is the unit weight of water, $c_{v}$ is the consolidation coefficient, which obtained by the Taylor method, and $m_{v}$ is the coefficient of volumetric change.

Note that the k-values obtained by this method agree with those obtained by the falling head percolation test for sand contents less than 50 % [26].

### 2.3 Sample preparation and testing

This study could not obtain intact, undisturbed samples from the field with various mixtures of sand, densities, and saturations for laboratory examination. As a result, test specimens consisted of hand-mixed blends of sand and clay. Standard compaction at various moisture contents produced the desired range of densities. The procedures shown below achieved the goal of forming homogeneous mixed samples. The methodology proposed by Monkul & Odzen [71] and later modified by Lupogo [72], produced satisfactory specimens. The procedure includes the following steps.

1 Calculate the required dry weights of sand and clay (based on the required sand percentage [Eq. (2)]) where the sand percentage (Fs) is the sand's weight divided by the solids' total weight.

(2) $$Fs,\%=\frac{ws}{wc+ws}*100$$

where,

*Fs*: percentage of sand (%)

*ws*: weight of sand in the mixture

*wc*: Weight of fine materials in the mixture.

2 For 10–15 min, mix dry sand and fine materials (dry clay) until the mixture appears homogeneous.
3 Add the required amount of water to the mixture.
4 Isolate and seal the mixture for 24 h for uniform moisture distribution.
5 Remix the soil for 15 min to ensure the sand grains distribute evenly.
6 Spoon the mixture mass into the oedometer mold, trim the top surface, and apply static pressure to achieve the desired unit weight.

According to Lupogo [72], during the preparation of samples for this method, the sample have excessive or low moisture, which will change the target unit weight, which will lead to a difficulty in controlling the water content, fine material content, sand, and unit weight of the specimen. Due to this, the sample formation method was modified.

**Fig. 4:**
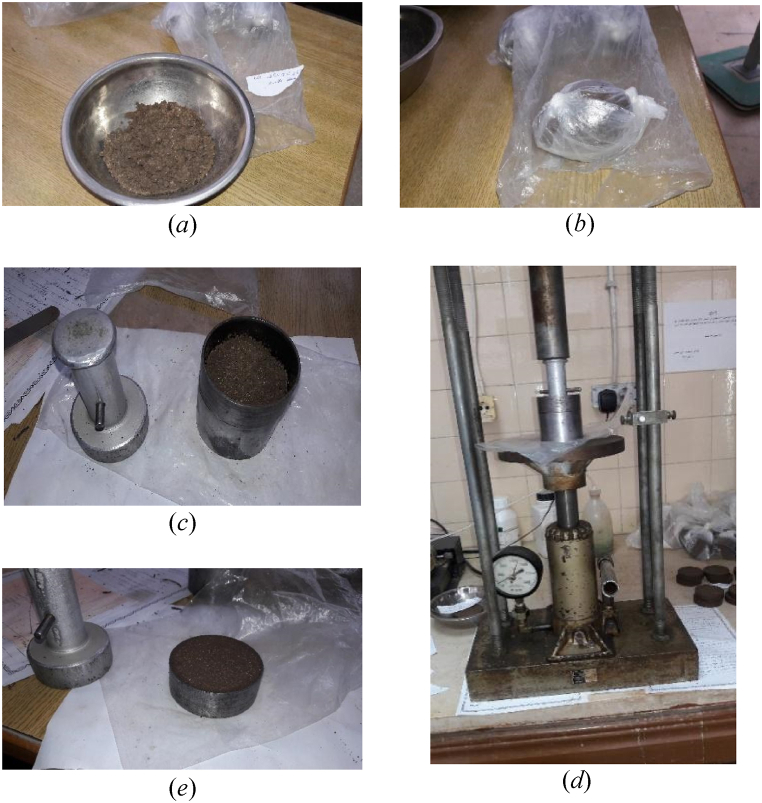
Images of the updated sample preparation procedure: (*a*) mixing sand and expansive clay; (*b*) enclosing the sample in plastic bags; (*c*) setting it within the ring; (*d*) using a hydraulic piston to apply static pressure; and (*e*) displaying the finished specimen.

The size of the sample mixture was reduced from 6 kg soil as Monkul & Odzen [71] did, to small quantities equal to the individual sample. As a result, there was better control of the void ratio, moisture content, fine material, and sand content in the mixed specimen. Fig. 4 depicts the updated procedure for preparing samples in the laboratory. Initially, a uniform mixture of sand and clay soils, as shown in Fig. 4(a), was created. To ensure even moisture distribution, this mixture was kept in an isolated plastic bag for 24 h, as shown in Fig. 4(b). Following that, the prepared mass was poured into a mold that matched the dimensions of the oedometer ring, as shown in Fig. 4(c). Using a hydraulic jack to apply static pressure, the specimen was compressed to the intended unit weight, as shown in Fig. 4(d). After that, the completed specimen, as shown in Fig. 4(e), was put inside the oedometer frame.

Table 7 depicts the initial conditions of the degree of saturation and the dry unit weight of the prepared samples. For each percentage of sand, 16 samples were prepared with four different initial dry unit weights, and each initial dry unit weight had four different degrees of saturation.

**Table 7:** The values for the initial states of the dry unit weight and the degree of saturation of the prepared samples.

*Fs i* (%)	$\gamma_{d,1}$; $\gamma_{d,2}$; $\gamma_{d,3}$; $\gamma_{d,4}\;\left(kN/m^{3}\right)$	*SR*_*1*_; *SR*_*2*_; *SR*_*3*_; *SR*_*4*_ (%)
0	13.28; 13.95; 14.63; 15.30	75; 80; 90; 100
10	14.02; 14.66; 15.30; 15.95	65; 75; 90; 100
20	14.30; 14.79; 15.30; 16.00	60; 75; 88; 100
30	15.30; 15.95; 16.60; 17.25	60; 75; 88; 100
40	15.30; 16.02; 16.73; 17.45	45; 75; 88; 100
50	15.30; 16.35; 17.40; 18.45	50; 75; 85; 100

**Fig. 5:**
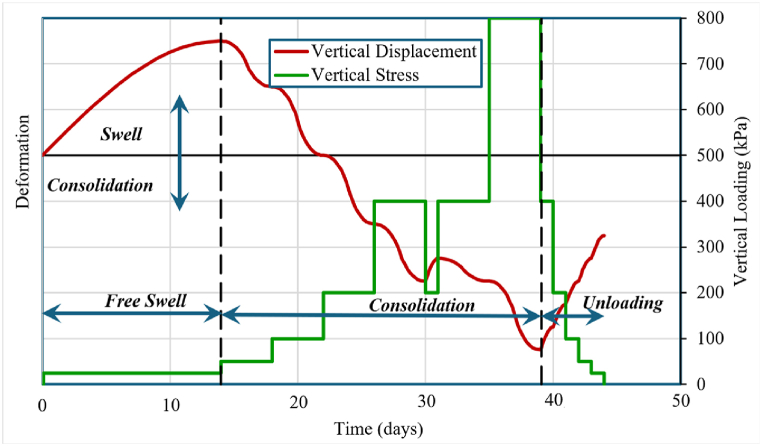
The odometer experiment testing sequence.

Fig. 5 depicts the odometer experiment testing sequence, which is as follows.

1. Prepare specimen
2. Place in oedometer apply 25 kPa
3. Allow to swell for 1–2 weeks until its deformation reaches equilibrium.
4. Implement the consolidation load sequence as follows: 50, 100, 200, 400, (200), 400, 800, (400), (200), (100), (50), (25)
a) Duration is to t_100_, about 48 h for loading, 24 h unloading.
b) Total duration of consolidation test is 1–2 months.
5. Remove specimen, measure moisture content.
6. Calculate $C_{c}$, $C_{r}$, $c_{v}$ based on log time at t_90_

### 2.4 Symbolic regression

Symbolic regression is a machine learning algorithm that finds mathematical equations or formulas that describe and fit data relationships optimally. It enables the modeling of complex relationships and the development of predictive equations without assuming anything about the underlying mathematical form. Symbolic regression outperforms Multiple Linear Regression (MLR), which is limited to linear relationships between variables, in capturing complex nonlinear relationships. Symbolic regression (*SR*) is widely applied in civil engineering [[73], [74], [75], [76], [77], [78], [79], [80], [81]]. Notably, symbolic regression equations outperformed traditional formulas in some cases [74,78,79]. In this study, the EUREQA software is used to discover latent patterns and relationships in the dataset and to generate equations to predict $C_{c}$ and $C_{r}$ for the studied soil in terms of the initial void ratio ($e_{0}$), the initial degree of saturation (*SR*), the liquid limit (*LL*), and the sand percentage (*Fs*) in the clay sample [82].

**Fig. 6:**
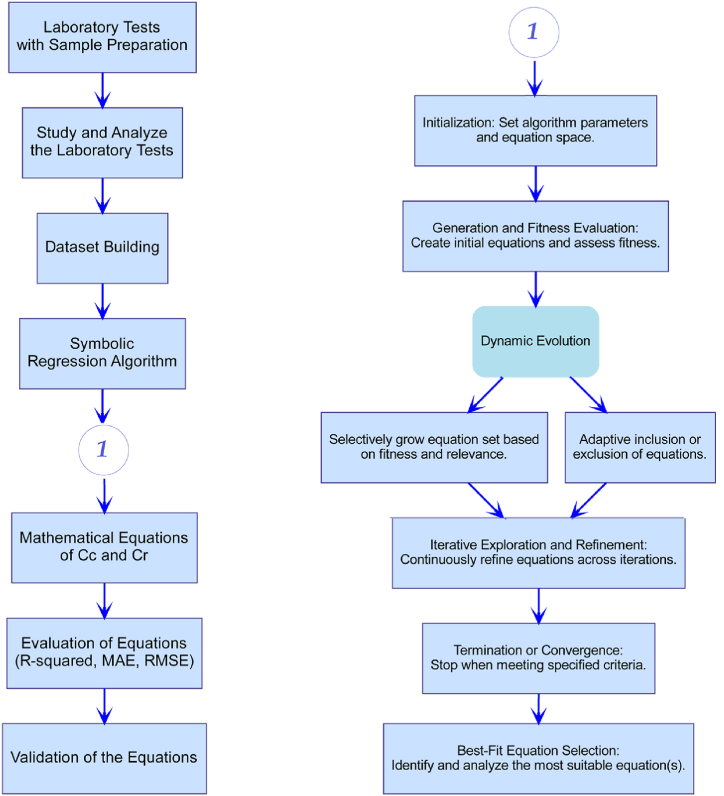
Workflow for laboratory processes and symbolic regression (symbolic regression algorithm is represented by 1).

The Symbolic Regression Algorithm's application in conjunction with a laboratory process is demonstrated through a flowchart (Fig. 6). Sample preparation and other laboratory tests are the first step. These tests are then studied and analyzed, and finally, a dataset is built. Afterwards, the process proceeds to the Symbolic Regression Algorithm, encompassing initialization, generation, fitness assessment, dynamic evolution, iterative exploration and refinement, termination or convergence, and ultimately, the identification of the optimal equation(s). The process then moves on to the evaluation and validation of the mathematical equations that were produced during the process.

In order to initiate the symbolic regression process, EUREQA first defines the equation space and configures the algorithm parameters, as illustrated in Fig. 6. First, a small set of equations is evaluated in order to determine their fitness to the given dataset. By adding and removing equations dynamically according to their relevance and fitness, the algorithm grows and enhances this set. The technique can iteratively explore improve equations by subjecting equations with high fitness to adaptive evolutionary mechanisms. By focusing computing resources on equations that show promise, EUREQA modifies the population of its equations. This dynamic evolution is repeated iteratively until convergence or the satisfaction of predefined termination criteria. Ultimately, the best-fit equation or equations are selected by EUREQA for analysis and validation of their accuracy in capturing the underlying patterns in the dataset.

### 2.5 Evaluation of models

Statistical indices were used to assess the predictive model's quality. The mathematical expressions for coefficient of determination (R^2^), root mean square error (RMSE), and mean percentage error (MAE) are Eqs. (3), (4), (5), respectively [[83], [84], [85]].

(3) $$R^{2}={\left(\frac{\sum_{i=1}^{n}\left(z_{i}-\bar{z}\right)\left(z_{i}^{\prime }-\bar{z^{\prime }}\right)}{\left(\sqrt{\sum_{i=1}^{n}{\left(z_{i}-\bar{z}\right)}^{2}}\right)\left(\sqrt{\sum_{i=1}^{n}{\left(z_{i}^{\prime }-\bar{z^{\prime }}\right)}^{2}}\right)}\right)}^{2}$$

(4) $$RMSE=\sqrt{\frac{\sum_{i=1}^{n}{\left(z_{i}^{\prime }-z_{i}\right)}^{2}}{n}}$$

(5) $$MAE\;\left(\%\right)=\frac{1}{n}\sum_{i=1}^{n}\left|\left(z_{i}^{\prime }-z_{i}\right)\right|\times 100$$

where n is the total number of data points, $\bar{z}$ is the average of the set z, and z and z′ stand for the true and predicted values, respectively. The proportion of the dependent variable's variance that the independent variables can predict is expressed by the coefficient of determination. It has a value between 0 and 1 and is the correlation coefficient (R) squared. If there is no correlation between the independent and dependent variables, the R^2^ value is 0., whereas when it is 1, the dependent variable can be accurately estimated. RMSE is used to express the standard deviation of differences between predicted and observed values. It is expressed as the square root of the mean of all squared errors. It denotes the absolute fit of observed data points to predicted values, whereas R^2^ denotes the relative fit of data points. The lower the value of RMSE, the better the fit and response of the predictive model. MAE calculates the percentage-based size of the error and displays how closely the predicted value matches the actual value. However, for zero or almost-zero actual values, it returns infinite or undefined values, which is regarded as a drawback [86]. Chai & Draxler [87] demonstrated that the RMSE has a clear measuring capacity. As a result, the combination of MAE and RMSE provides a more confident assessment of predictive model performance.

**Fig. 7:**
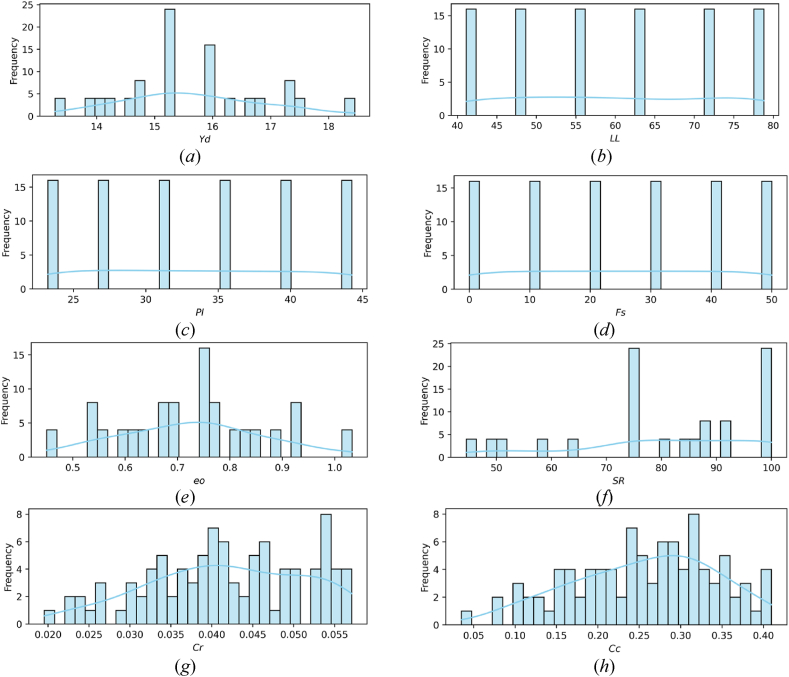
Histogram-illustrated parameter distribution: (*a*) $\gamma_{d}$, (*b*) *LL,* (*c*) *PI*, (*d*) *Fs*, (*e*) $e_{o}$, (*f*) *SR*, (*g*) $C_{r}$ , (*h*) $C_{c}$.

The statistical characteristics of the parameters under consideration are shown in Table 8. Additionally, the distribution of these parameters is depicted using histograms in Fig. 7(a–h). For example: Fig. 7(a) shows the distribution of the variable $\gamma_{d}$ over the range of 13–19 $\text{kN}/m^{3}$, with the x-axis displaying $\gamma_{d}$ values and the y-axis indicating the number of occurrences inside each bin. Notably, the bin centered at $\gamma_{d}$ = 15.3 $\text{kN}/m^{3}$ has the highest frequency, with roughly 24 occurrences, followed by the bin at $\gamma_{d}$ = 15.95 $\text{kN}/m^{3}$, which has about 16 occurrences. This suggests a concentration of $\gamma_{d}$ values near these central areas. Furthermore, a smooth density curve is superimposed on the histogram to provide a visual depiction of the data's probability distribution function. This curve peaks at $\gamma_{d}$ = 15.3 $\text{kN}/m^{3}$, which corresponds to the highest frequency bin, and has a secondary peak near $\gamma_{d}$ = 15.95 $\text{kN}/m^{3}$.The density curve's shape is consistent with the overall distribution of the histogram, emphasizing the concentration of data points around these values while indicating lower frequencies at the distribution's extremities (13.3 and 18.45 $\text{kN}/m^{3}$). The similarity of other histograms in Fig. 7(b–h) helps to provide similar observations.This thorough representation helps to comprehend the central trends and dispersion of the variables within the provided ranges.

**Table 8:** The statistical characteristics of the parameters under consideration.

	*LL*	*Fs*	*e*_*0*_	*SR*	$\gamma_{d}$	$C_{c}$	$C_{r}$
count	96	96	96	96	96	96	96
mean	59.95	25	0.728	80.4	15.65	0.2553	0.04183
std	13.08	17.17	0.139	16.76	1.234	0.08647	0.00956
min	41	0	0.45	45	13.28	0.035	0.01953
25 %	48.6	10	0.631	75	14.765	0.1954	0.0349
50 %	59.55	25	0.75	82.75	15.3	0.2652	0.0418
75 %	72.1	40	0.823	93.48	16.412	0.315	0.0496
max	79	50	1.033	100	18.45	0.4095	0.0571

## 3 Results and discussion

### 3.1 Impact of initial dry unit weight

**Fig. 8:**
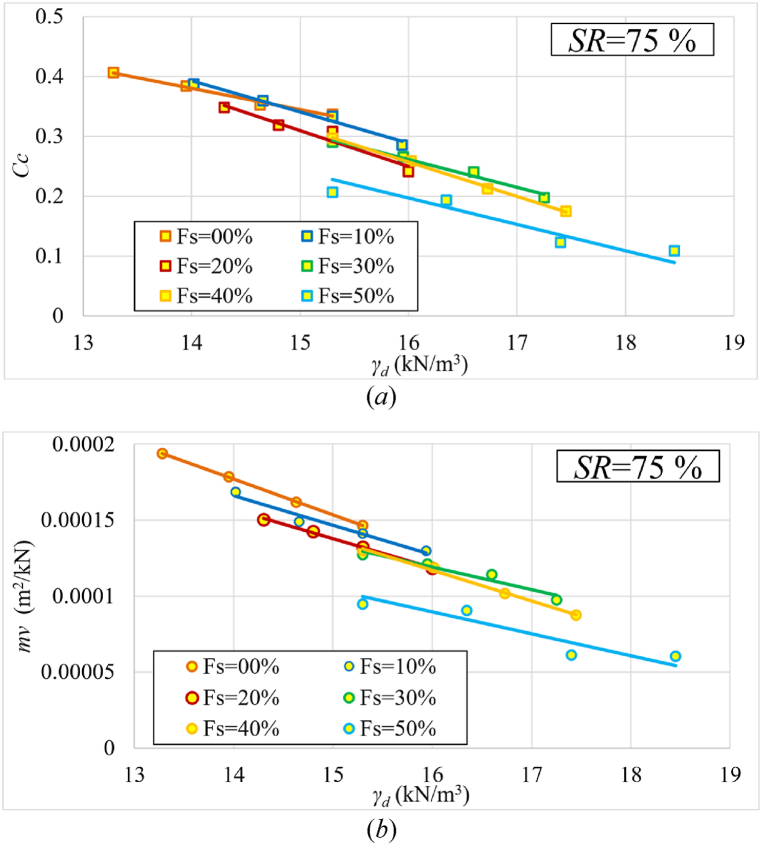
Changes in **(*a*)**$C_{c}$ and **(*b*)**$m_{v}$ as a function of initial dry unit weight ($\gamma_{d})$ for various sand percentages at 75 % initial saturation level.

The effect of initial dry unit weight was investigated by setting the initial saturation level at 75 % for each sample with prescribed percentages of added sand and varying densities. Fig. 8(a and b) depicts the change in compression index ($C_{c}$) and coefficient of volume compressibility ($m_{v}$) with initial dry unit weight of samples for each sand percentage added.

Fig. 8 (a) and (b) shows that $C_{c}$ and $m_{v}$ decreased as the dry unit weight increased for each percentage of sand. It is also noticed that there is a decrease in these values as the percentage of sand increases, which is consistent with [9].

The addition of sand to expansive clayey soils significantly increases the dry unit weight of the soil mixture, decreasing soil compressibility. A 50 % sand content increases the dry unit weight to 18.4 kN/m³ from 15.3 kN/m³ without sand. This is due to the sand's role in reducing the void ratio of the soil mix and becoming less water-holding, as demonstrated by the study of [22]. As a result, both the compression index ($C_{c}$) and the coefficient of volume compressibility ($m_{v}$) show significant reduction. $C_{c}$ decreases by 70 %, while $m_{v}$ decreases by 40 %. The consistent trend in dry unit weight on both $C_{c}$ and $m_{v}$ demonstrates the positive impact of dry unit weight on both $C_{c}$ and $m_{v}$ across different sand percentages.

**Fig. 9:**
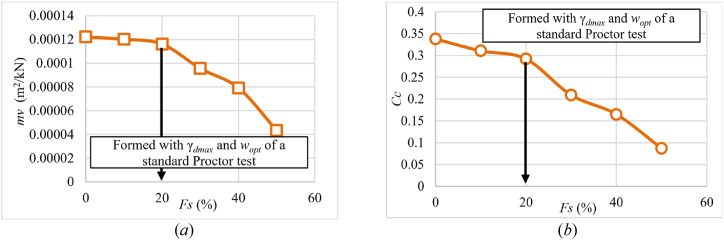
**(*a*)** Variation of $m_{v}$; **(*b*)** Variation of $C_{c}$ as a function of percentage of added sand for samples formed with optimum moisture and maximum dry unit weight of a standard Proctor test.

Due to the importance of proctor tests, the authors conducted oedometric tests to show the real behavior of improved soils in the field. Fig. 9 shows the effect of sand percentage on the compression parameters with optimal water content and maximum dry unit weight. The decrease in both $C_{c}$ and $m_{v}$ became more severe after the 20 % of added sand, as shown in Fig. 9(a) and (b). The reason for this was that as the percentage of sand increased, the unit weight of the soil improved and became more amenable to compaction; as a result, the maximum dry unit weight increased and became less capable of holding water.

**Fig. 10:**
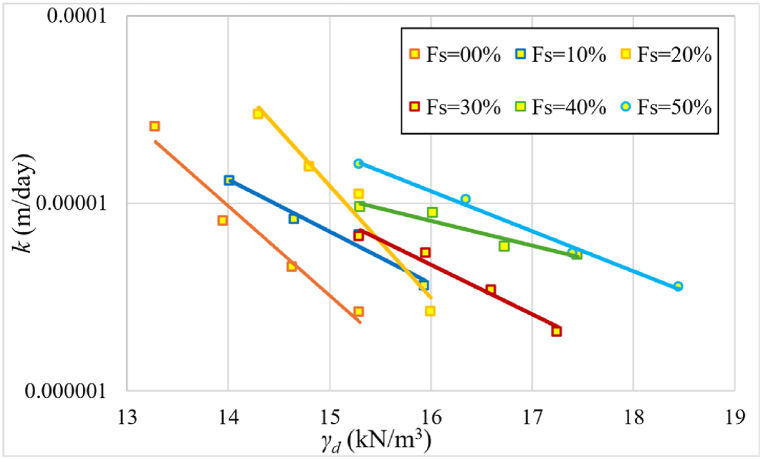
Change in permeability (*k*) values as a function of $\gamma_{d}$ for different percentages of added sand to the samples at 75 % initial saturation level.

Fig. 10 shows the change in permeability values as a function of initial dry unit weight for samples with varying percentages of sand content.

**Fig. 11:**
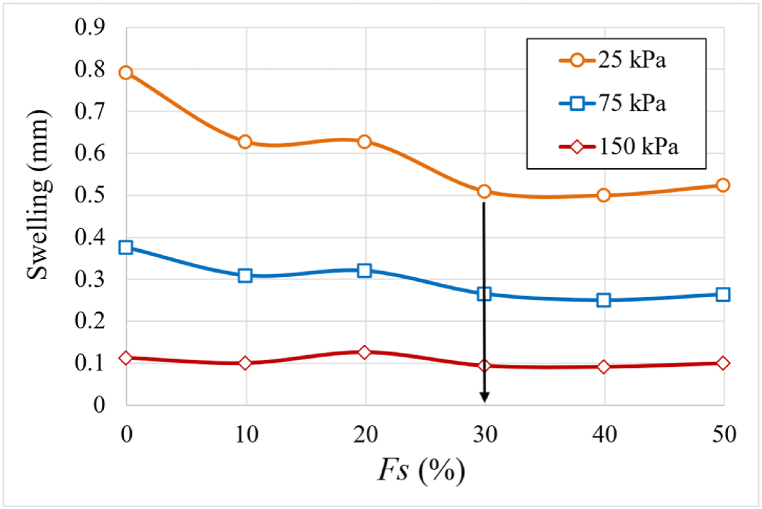
The relationship, at three distinct applied pressures of 25, 75, and 150 kPa, between the swelling and the percentage of sand for formed samples according to the standard Proctor.

Permeability decreases with increasing dry unit weight, as seen in Fig. 10, which is consistent with [9]. There are two sets of curves. Curves with sand percentages ranging from 0 % to 20 % are included in the first group. The permeability coefficient increases by 250 % at a dry unit weight of 15 kN/m³. At a dry unit weight of 17 kN/m³, the permeability coefficient rises by 130 % in the second group, where the sand percentage varies from 30 to 50 %. This can be due to the significant reduction in swelling after adding 30 % sand, as the sand begins to influence the samples' behavior. The ideal sand content is 30 %, which balances dry unit weight and water retention. More research into the texture of samples at this percentage is required to understand the composition and behavior within the specimens. Notably, the 30 % sand addition resulted in the lowest permeability coefficient due to the lower swelling at this percentage as shown in Fig. 11.

Fig. 11 depicts the effect of different sand percentages on soil swelling under various applied pressures. In general, swelling diminishes as the sand percentage rises to 10 %, with the decrease being more evident at low applied pressure and becoming less noticeable as thethe stress increases, maintaining nearly constant until 20 %. The lowest swelling can be seen at 30 % sand content, followed by a modest increase in swelling.

**Fig. 12:**
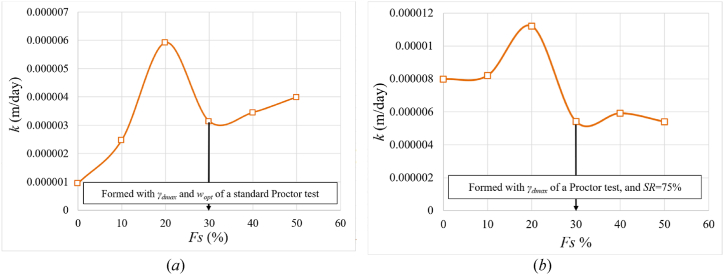
Variation of the permeability values as a function of the percentage of sand (*Fs*); **(*a*)** samples formed according to the standard Proctor's optimal moisture and maximum dry unit weight; **(*b*)** samples formed according to the standard Proctor's maximum dry unit weight and at an initial saturation degree of 75 %.

As shown In Fig. 11, for applied pressure 150 kPa where $m_{v}$ and k is calculated at, the sample shows swelling during saturation up to 20 % added sand. This swelling expands the voids in the sample, thus increases permeability. However, the least swelling occurs at 30 % added sand, leading to the lowest permeability. Although coarse materials typically enhance permeability, once 30 % or more coarse materials (sand) are added, there's a notable reduction in both swelling and void size. Consequently, permeability undergoes a significant decrease, as demonstrated in Fig. 12(a) and (b).

**Fig. 13:**
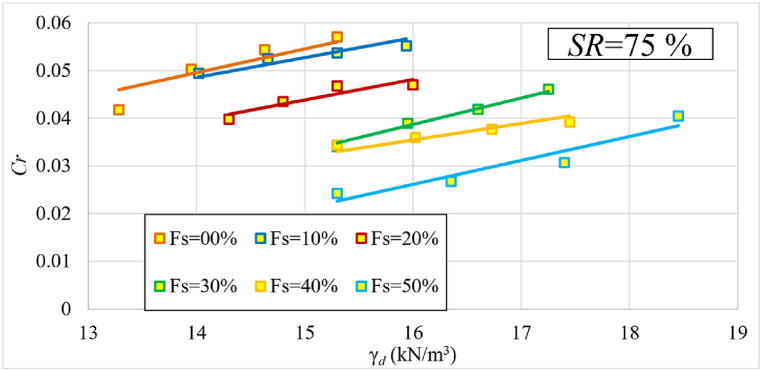
The change in recompression index ($C_{r}$) values as a function of $\gamma_{d}$ in terms of each percentage of added sand at 75 % initial saturation level.

Fig. 13 depicts the change in recompression index ($C_{r}$) values as a function of the samples’ initial dry unit weights in terms of each percentage of sand. The trendline of $C_{r}$ versus $\gamma_{d}$ across the entire dataset is consistent with the findings from previous research, such as [10,24,29]. Fig. 13 shows that $C_{r}$ values decrease as the sand percentage increases. At a sand percentage of 50 % and a dry unit weight of 15.3 kN/m³, $C_{r}$ reaches its lowest value, decreasing by approximately 52 % as the sand percentage rises from 0 to 50 %. Conversely, $C_{r}$ values increase with higher dry unit weights for each sand percentage. It is noteworthy that the trend of the curves, represented by the tangent of the straight lines, remains relatively consistent in all cases. The reason for this phenomena is because increasing the sand percentage reduces the clay content in the sample, increasing its stiffness and making it less susceptible to water absorption, resulting in lower $C_{r}$ values after unloading. Meanwhile, for the same sand percentages, raising the dry unit weight improves stiffness but increases the amount of clay, resulting in increased swelling after unloading. This is why $C_{r}$ values increase with increasing dry unit weight at the same sand content.

**Fig. 14:**
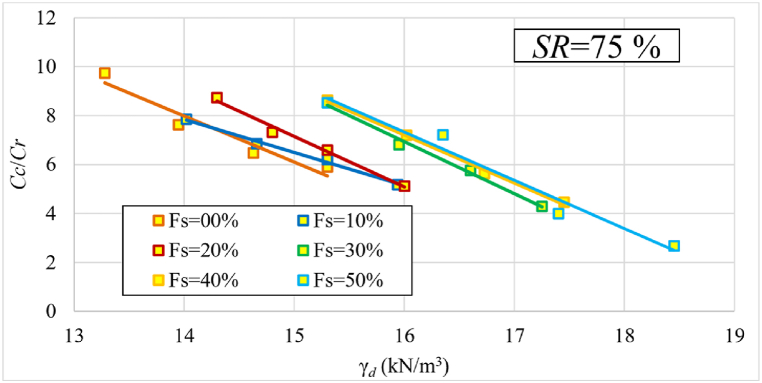
The change in ratio ($C_{c}$/ $C_{r}$) as a function of $\gamma_{d}$ at 75 % initial saturation level.

Fig. 14 illustrates the change in ratio ($C_{c}$/ $C_{r}$) values as a function of samples' initial dry unit weight ($\gamma_{d}$) in terms of each percentage of sand. It reveals that as $\gamma_{d}$ increases, the ratio of $C_{c}$/ $C_{r}$ decreases. It is also worth noting that after adding 30 % sand, the sand's behavior becomes dominant as the curves merge into one bundle. The $C_{c}$/ $C_{r}$ ratio ranged between (2.8–9.7) depending on the percentage of sand and initial dry unit weight.

### 3.2 Impact of initial saturation level

**Fig. 15:**
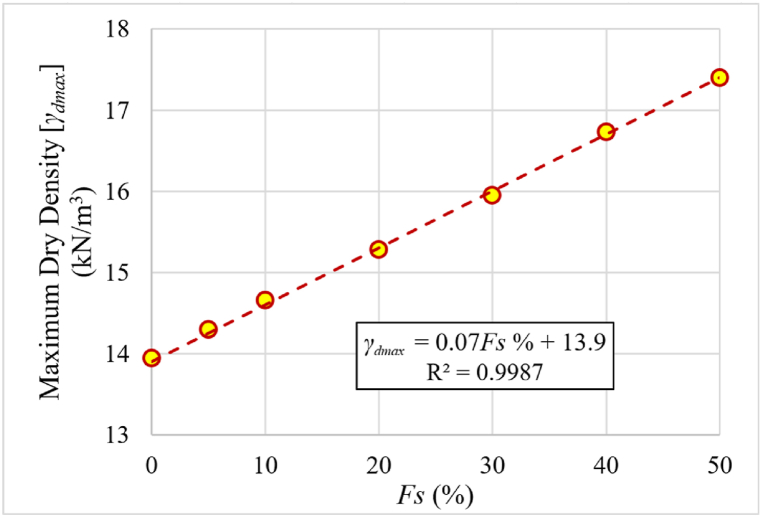
Relationship between percentage of added sand and maximum dry unit weight.

**Fig. 16:**
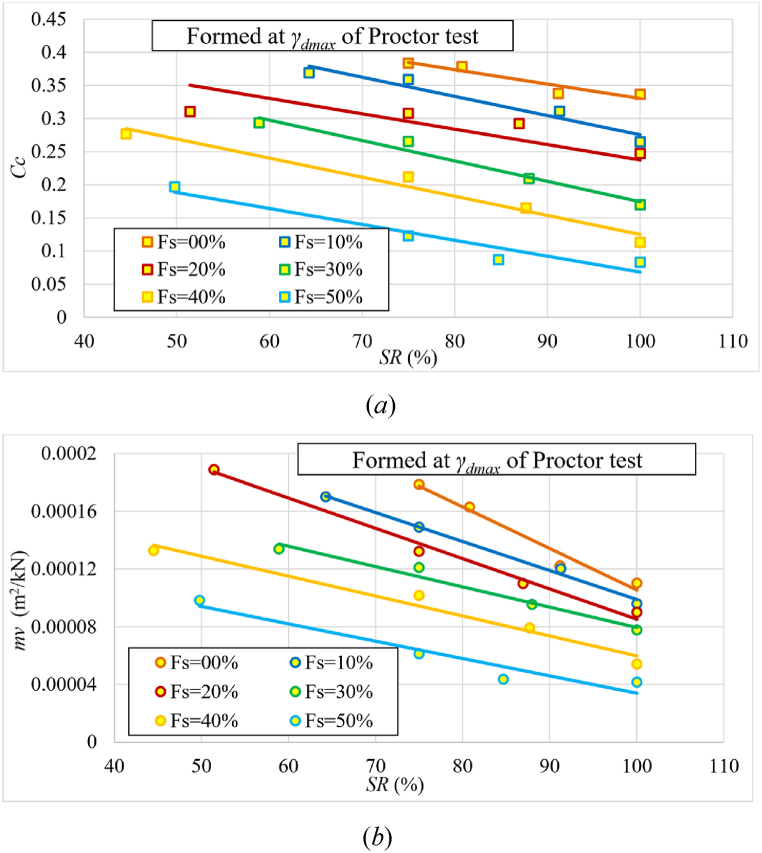
The changes in **(*a*)**$C_{c}$ and **(*b*)**$m_{v}$ as a function of the degree of saturation (*SR*) for various sand percentages of samples formed at $\gamma_{dmax\;}$ of standard Proctor test.

The changes in the initial degree of saturation for each of the samples mixed with different percentages of added sand were studied separately. The maximum dry unit weight determined by the standard Proctor test was set for each percentage of sand. Fig. 15 shows that as the sand percentage increases, so does the maximum dry unit weight. Fig. 16 (a) and (b) depict the alteration in $C_{c}$ and $m_{v}$ as a function of the degree of saturation (*SR*) for various sand percentages.

Both $C_{c}$ and $m_{v}$ decrease with increasing initial saturation and sand percentage in the sample (Fig. 16). It's worth mentioning that in many previous studies, the focus was primarily on natural moisture levels rather than the degree of saturation. However, our dataset's trendline of $C_{c}$ with the $w_{n}$ aligns with findings from earlier research, such as [25,29,39,42]

**Fig. 17:**
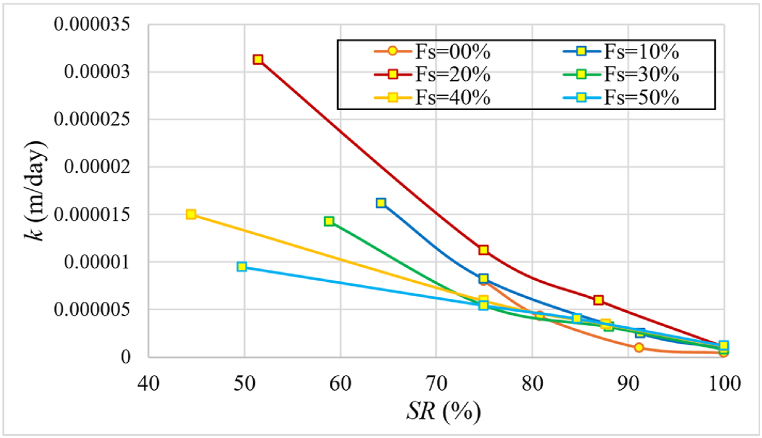
Changes of permeability (*k*) with the initial degree of saturation (*SR*) for various sand percentages of samples formed at $\gamma_{dmax\;}$ of standard Proctor test.

Fig. 17 depicts how the permeability changes with the initial saturation. The saturated permeability decreases as the initial saturation increases. This is because higher initial saturation results in reduced swelling, which in turn leads to smaller void sizes within the sample, thereby decreasing the permeability. It was also observed that the saturated permeability increased with increasing percentage of sand up to 20 % and then it decreased. This is because after the 20 % of added sand, the soil became more compact and less able to hold water, and volume changes became smaller.

**Fig. 18:**
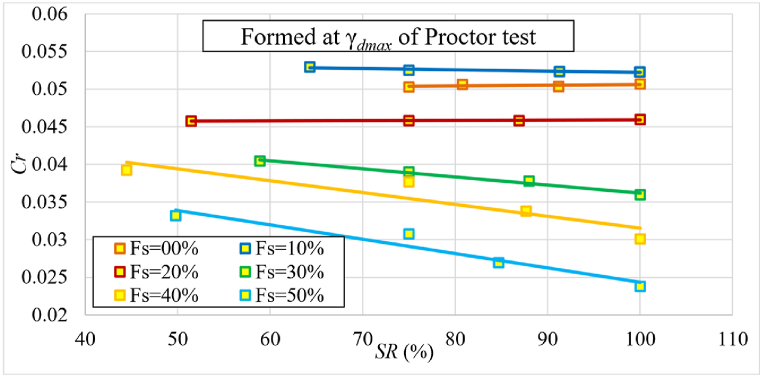
The change of the recompression index ($C_{r}$) as a function of the initial degree of saturation for various sand percentages of samples formed at $\gamma_{dmax\;}$ of standard Proctor test.

Fig. 18 shows the change of the recompression index ($C_{r}$) as a function of the initial saturation for various percentages of sand. Fig. 18 shows that $C_{r}$ decreased slightly as the initial degree of saturation increased up to the 20 % of sand, and the decrease became noticeable after 20 %. It was also noticeable that the value of $C_{r}$ decreased as the percentage of sand increased, especially after the ratio of 20 %. The reason for this is that as sand becomes more dominant, suction stresses decrease and thus the ability to hold water decreases. It is worth noting that many previous studies primarily focused on natural moisture levels rather than the degree of saturation. Nevertheless, the trendline of $C_{r}$ with $w_{n}$ in our dataset aligns with findings from earlier research, such as [10,24,29]

### 3.3 Symbolic regression to predict $C_{c}$ and $C_{r}$

#### 3.3.1 Proposed equations of $C_{c}$

**Fig. 19:**
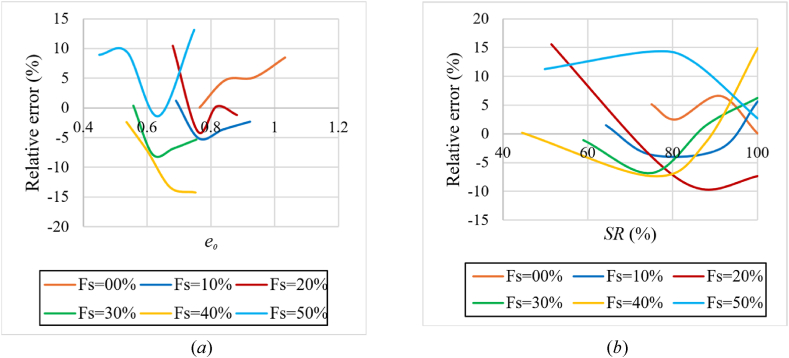
Relative error in predicting the compression index ($C_{c}$) using Equation (6)**(*a*)** relative error according to the initial void ratio**; (*b*)** relative error according to the initial degree of saturation for various percentages of added sand.

For the prediction of compression index ($C_{c}$), equations (6), (7), (8) are formulated using the EUREQA software. These equations are valid if the percentage of sand in the sample does not exceed 50 %, the Atterberg limits are close to line A on Casagrande's plasticity chart, and the initial degree of saturation is greater than 50 %. The liquid limit is between (40–80 %) and the applied loading during saturation is 25 kPa. The relative error under these conditions was no more than 10 % [Relative error (%)=(Eq. (6) value – experimental value)*100/experimental value]. Except in small regions that exceed it and reach a 15 % error rate, as shown in Fig. 19(a) and (b). It is worth noting that the proposed equations has a higher R-squared value than the equations in Table 2.

(6) $$C_{c}=0.243+0.381*e_{0}-0.00195*Fs-0.00267*\mathrm{SR}\;\left(R^{2}=0.968;\;\text{RMSE}=0.015;\;\text{MAE}=1.14\%\right)$$

(7) $$C_{c}=0.1232+0.3859*e_{0}-1.718*10^{-5}*SR^{2}-3.641*10^{-5}*Fs^{2}\;\left(R^{2}=0.983;\;\text{RMSE}=0.0109;\;\text{MAE}=0.72\%\right)$$

(8) $$C_{c}=0.475+0.00409*\text{LL}+0.00179*\text{LL}*e_{0}+5.5*10^{-5}*LL*Fs-0.0319*\gamma_{d}-1.72*10^{-5}*SR^{2}\;\left(R^{2}=0.985;\;\text{RMSE}=0.0103;\;\text{MAE}=0.6673\%\right)$$

where:

$C_{c}$: Compression index

$e_{0}$: Initial void ratio: $e_{0}=\frac{\gamma_{s}}{\gamma_{d}}-1$.

$\gamma_{d\;}\text{:}$ Dry unit weight kN/m^3^

$\gamma_{s}$: Unit weight of solids kN/m^3^

SR: Initial degree of saturation %

Fs: Percentage of added sand %

Table 9 shows a comparison of MAE and RMSE metrics with the equations listed in Table 2, using the data from this study. The equations in this study clearly outperform the others. Notably, the metrics of Herrero (2) [25], Azzouz et al. [29], Koppula [39], and Cozzolino [37] equations are the closest to those of this study, as shown in Table 9.

**Table 9:** MAE and RMSE metrics for $C_{c}$ equations in current and previous studies using the dataset used in this study.

Equation	MAE (%)	RMSE
This study [Eq. (6)]	1.14	0.015
This study [Eq. (7)]	0.72	0.011
This study [Eq. (8)]	0.67	0.010
Herrero 1983 (2) [25]	4.46	0.055
Azoz et al., 1976 [29]	5.26	0.065
Koppula 1981 [39]	8.05	0.098
Cozzolino 1961 [37]	9.09	0.103
Skempton 1944 [33]	9.93	0.114
Nagaraj and Srinivasa 1983 [34]	10.75	0.121
Herrero 1983 (1) [25]	11.86	0.144
Houghs 1957 [35]	12.29	0.135
Herrero 1980 [26]	12.45	0.140
Bowles 1989 [38]	13.24	0.148
Terzaghi and Peck 1967 [36]	19.43	0.210
Wroth and Wood 1978 [40]	19.62	0.207
Mohammadzadeh et al., 2019 [41]	58.40	0.593

**Fig. 20:**
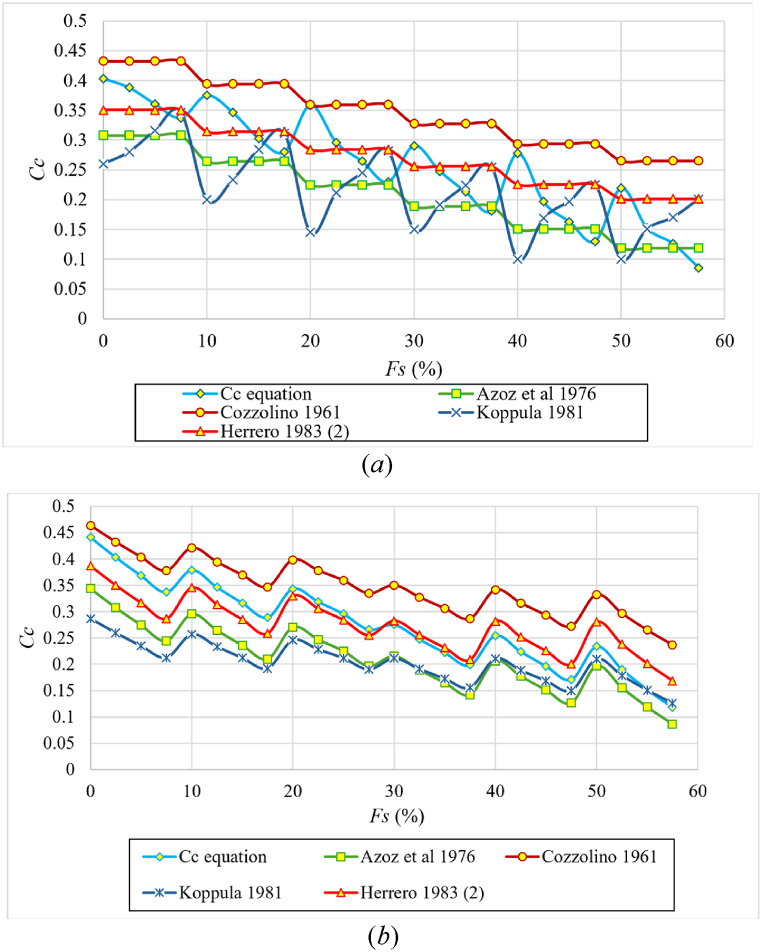
Comparison of the proposed Eq. (6) with the best prediction equations mentioned in the references according to the percentage of sand for **(*a*)** different initial dry densities, and **(*b*)** different initial degrees of saturation.

Fig. 20 (a) and (b) compares the $C_{c}$ results of proposed Eq. (6) to Herrero (2) [25], Azzouz et al. [29], Koppula [39], and Cozzolino [37] equations for **different degrees of initial saturation and different initial dry densities** for each percentage of sand, respectevely. It should be noted that the equations are good predict the effect of **initial dry densities. However** none of the equations accounted for the effect of the **initial degree of saturation** on the compression index $C_{c}$ or correctly predicted its behavior as for Koppula equation.

#### 3.3.2 Proposed equations of $C_{r}$

**Fig. 21:**
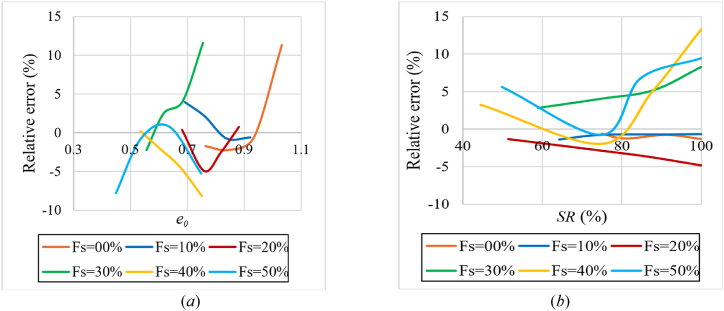
Relative error in predicting the recompression index ($C_{r}$) using equation (10); **(*a*)** relative error according to the initial void ratio; **(*b*)** relative error according to the initial degree of saturation for various percentages of added sand.

For the prediction of recompression index ($C_{r}$), equations (9), (10) are formulated using the EUREQA software. These equations are valid if the percentage of sand in the sample does not exceed 50 %, the Atterberg limits are close to line A on Casagrande's plasticity chart, and the initial degree of saturation is greater than 50 %. The relative error under these conditions was no more than 10 % [Relative error (%) = (Eq. (9) value – experimental value)*100/experimental value], as shown in Fig. 21(a) and (b). It is worth noting that the proposed equations has a higher R-squared value than the equations in Table 3.

(9) $$C_{r}=0.00289*LL+0.00171*Fs\;-\;0.0401*e_{0}\;-\;3.12*10^{-6}*SR*Fs\;-\;0.139\;\left(R^{2}=0.956;\;\text{RMSE}=2.03*10^{-3};\;\text{MAE}=0.155\%\right)$$

(10) $$C_{r}=0.00356*LL+0.00228*Fs\;-\;0.0328*e_{0}\;-\;6.725*10^{-8}*SR*{Fs}^{2}\;-\;0.2\;\left(R^{2}=0.967;\;\text{RMSE}=1.76*10^{-3};\;\text{MAE}=0.125\%\right)$$

Table 10 shows a comparison of metrics with the equations listed in Table 3, using the data from this study. The equations in this study clearly outperform the others. Notably, the MAE and RMSE metrics of Sinan (1,2 and 3) [10], Kordnaeij et al. [24], Nakase et al. [52], and Gunduz and Arman [51] equations are the closest to those of this study, as shown in Table 10.

**Table 10:** MAE and RMSE metrics for $C_{r}$ equations in current and previous studies using the dataset used in this study.

Equation	MAE (%)	RMSE
This study [Eq. (9)]	0.16	0.00203
This study [Eq. (10)]	0.13	0.00176
Sinan. 2009 (1) [10]	0.66	0.00791
Sinan. 2009 (2) [10]	0.98	0.01171
Sinan. 2009 (4) [10]	1.14	0.01376
Kordnaeij et al., 2015 [24]	1.32	0.01658
Nakase et al., 1988 [52]	1.75	0.01826
Gunduz and Arman. 2007 [51]	1.79	0.02096
Sinan. 2009 (3) [10]	2.86	0.03010
Azzouz et al., 1976 (2) [29]	3.15	0.03278
Nagaraj and Murty. 1985 [53]	3.28	0.03413
Azzouz et al., 1976 (1) [29]	6.50	0.06756

**Fig. 22:**
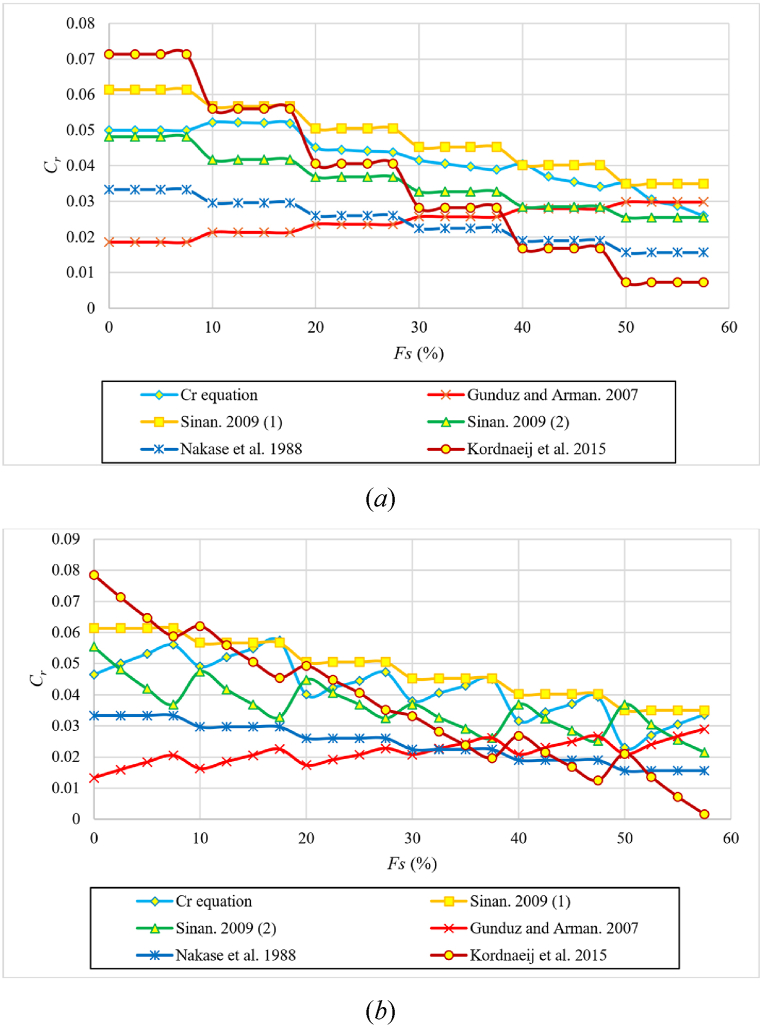
Comparison of the proposed Eq. (10) with the best prediction equations mentioned in the references according to the percentage of sand; **(*a*)** different initial dry densities; **(*b*)** different initial degrees of saturation.

Fig. 22 (a) and (b) compares the proposed Eq. (9) with the equations mentioned in previous research (Table 3) for **different degrees of initial saturation** and **different initial dry densities** for each percentage of sand, respectevely. It is noted from Fig. 22(a) that all equations do not account for the effect of the initial **degree of saturation** on the recompression index $C_{r}$. It is also noted from Fig. 22(b) that some equations did not have an effect on the **initial dry unit weight** on the recompression index $C_{r}$ or it did have an effect but in a wrong behavior.

#### 3.3.3 Validation of $C_{c}$ and $C_{r}$ equations

The validation was performed on the dataset FI-CLAY/14/856, which follows the TC304 naming guidelines [88]. For more statistical information about the dataset, see Ref. [42]. Lines in the dataset that were irrelevant to our study were removed, as were some outliers.

**Fig. 23:**
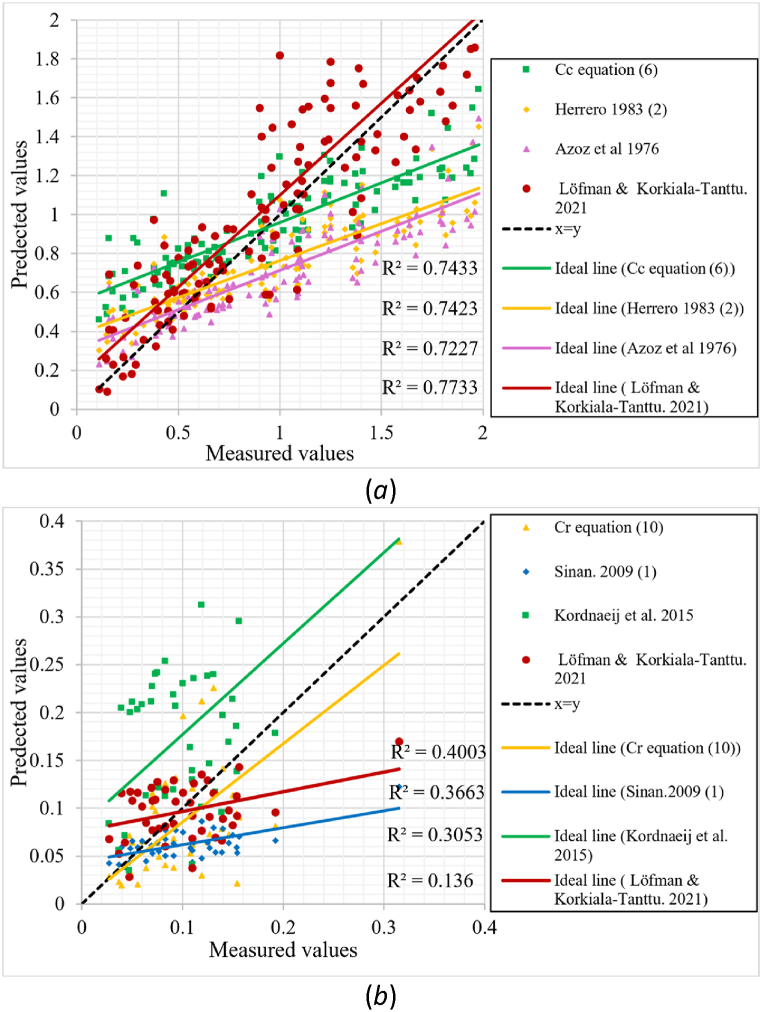
Comparing the actual and predicted values of $C_{c}$ and $C_{r}$ of the FI-CLAY/14/856 dataset; (*a*) $C_{c}$, (*b*) $C_{r}$.

Fig. 23(a) depicts an evaluation of the equations' prediction capacities for $C_{c}$. This chart compares the dataset's actual and predicted values with the best predictive equations proposed in this work, including the Herrero and Azzouz equations, as well as the Löfman & Korkiala-Tanttu equation, which was derived from the FI-CLAY/14/856 dataset. As seen in Fig. 23(a), all equations have identical R-squared values that exceed 0.7. The proposed equation (6) has an R-squared value of 0.743, similar to the Herrero equation. However, for the tested dataset, the Mean Absolute Error (MAE) and Root Mean Squared Error (RMSE) for the proposed $C_{c}$ equation are 26.8 and 0.326, respectively, compared to 30.3 and 0.3922 for the Herrero equation and 31.2 and 0.42 for the Azzouz equation. Furthermore, the proposed equation's ideal line is the closest to the x = y line and the ideal line of the Löfman & Korkiala-Tanttu equation shown in Table 2. The ideal line of the Löfman & Korkiala-Tanttu equation is the closest to the x = y line because this equation was derived from the same dataset, resulting in a MAE of 21.13 and an RMSE of 0.282.

An analysis of the predictive power of the equations for $C_{r}$ is shown in Fig. 23(b). It makes a comparison between the dataset's actual and predicted values as well as with more accurate equations from earlier research—more precisely, the Sinan and Kordnaeij et al. equations. The proposed equation (10) has the highest R-squared value, roughly 0.4, as seen in Fig. 23(b). On the other hand, the tested dataset's MAE and RMSE for the proposed $C_{r}$ equation (10) in this study are 4.41 and 0.0538, respectively, whereas the Löfman & Korkiala-Tanttu equation's values are 3.04 and 0.0484, the Sinan equation's values are 4.24 and 0.0587 and the Kordnaeij et al. equation's values are 8.38 and 0.106. It is also worth noting that the ideal line of the proposed equation is closest to the x = y line.

## 4 Conclusion

The impacts of sand content, initial dry unit weight, and degree of saturation are investigated in this study to provide significant insights into the compressibility behavior of expansive soils. Depending on the experimental results and Symbolic regression analysis of expansive clayey soil mixed with different percentages of sand, the results can be summarized as follows.

1. For each specific percentage of sand, both $C_{c}$ and $m_{v}$ decrease as the dry unit weight increases. There is also a decrease in these values as the percentage of sand increases, and a decrease becomes more severe after 20 % of added sand.
2. There is a significant reduction in permeability (*k*) as dry unit weight increases. The lowest permeability values are obtained at an added sand of 30 %.
3. The values of the recompression index ($C_{r}$) decrease as the percentage of sand increases, whereas the values of $C_{r}$ increase as the dry unit weight of each percentage of added sand increases.
4. As dry unit weight increases, the ratio $C_{c}$/ $C_{r}$ decreases. After adding 30 % sand, the sand's behavior takes over as the curves merge into one bundle. The ratio $C_{c}$/ $C_{r}$ values varied according to the sand and dry unit weight ratio between (2.8–9.7).
5. The compression index ($C_{c}$) and the coefficient of volume compressibility ($m_{v}$) decrease as the degree of saturation and the percentage of sand increase. As the percentage of added sand and the initial degree of saturation increase, both $C_{c}$ and $m_{v}$ decrease.
6. As the initial degree of saturation increases, the permeability decreases. It is also worth noting that the permeability increases with the percentage of sand up to 20 % and then decreases after that.
7. The recompression index, $C_{r}$, decreases slightly with increasing saturation until the percentage of sand reaches 20 %, at which point the decrease becomes noticeable. The value of $C_{r}$ decreases as the percentage of sand increases, especially after the ratio of 20 %.
8. Empirical Eqs. (5), (6), (7), (8), (9) are found and validated for $C_{c}$ and $C_{r}$ in the saturated state of the studied soil, in terms of initial void ratio ($e_{0}$), initial degree of saturation (*SR*), liquid limit (*LL*), and percentage of sand (*Fs*) in the clay sample.

The empirical equations (5), (6), (7), (8), (9) were thoroughly tested against the experimental data as well as the new dataset FI-CLAY/14/856 to validate their results. When the sand content is less than 50 %, the Atterberg limits are close to line A on Casagrande's plasticity chart, and the initial degree of saturation is greater than 50 %, the equations perform best. These requirements guarantee the predictive models' dependability and precision, giving engineers working in the field a useful tool. These equations might be improved through more study to make them applicable outside of these limits.

## Funding statement

This research received no external funding. Funding for open access granted by Szechenyi István University (SZE).

## Data availability statement

Data will be made available upon request.

## CRediT authorship contribution statement

**Ammar Alnmr:** Writing – review & editing, Writing – original draft, Visualization, Validation, Software, Methodology, Investigation, Formal analysis, Data curation, Conceptualization. **Rashad Alsirawan:** Writing – review & editing, Visualization, Methodology, Formal analysis. **Richard Ray:** Writing – review & editing, Validation, Supervision, Methodology, Conceptualization. **Mounzer Omran Alzawi:** Writing – review & editing, Visualization, Validation, Supervision, Methodology, Investigation, Data curation, Conceptualization.

## Declaration of generative AI and AI-assisted technologies in the writing process

During the revission of this work the author used ChatGPT in order to improve language and readability. After using this tool/service, the authors reviewed and edited the content as needed and take full responsibility for the content of the publication.

## Declaration of competing interest

The authors declare that they have no known competing financial interests or personal relationships that could have appeared to influence the work reported in this paper.
